# Multiscale Geometric Analysis Fusion-Based Unsupervised Change Detection in Remote Sensing Images via FLICM Model

**DOI:** 10.3390/e24020291

**Published:** 2022-02-18

**Authors:** Liangliang Li, Hongbing Ma, Zhenhong Jia

**Affiliations:** 1 Department of Electronic Engineering, Tsinghua University, Beijing 100084, China; leeliangliang@163.com; 2 College of Information Science and Engineering, Xinjiang University, Urumqi 830046, China; jzhh@xju.edu.cn

**Keywords:** remote sensing image, change detection, NSCT, FLICM, difference image

## Abstract

Remote sensing image change detection is widely used in land use and natural disaster detection. In order to improve the accuracy of change detection, a robust change detection method based on nonsubsampled contourlet transform (NSCT) fusion and fuzzy local information C-means clustering (FLICM) model is introduced in this paper. Firstly, the log-ratio and mean-ratio operators are used to generate the difference image (DI), respectively; then, the NSCT fusion model is utilized to fuse the two difference images, and one new DI is obtained. The fused DI can not only reflect the real change trend but also suppress the background. The FLICM is performed on the new DI to obtain the final change detection map. Four groups of homogeneous remote sensing images are selected for simulation experiments, and the experimental results demonstrate that the proposed homogeneous change detection method has a superior performance than other state-of-the-art algorithms.

## 1. Introduction

The application of remote sensing images is more and more extensive in the current research. These applications include image fusion [1,2,3,4,5,6], image classification [7,8,9,10,11], change detection [12,13,14,15,16,17], etc. In particular, remote sensing image change detection is to calculate the changed region from the images obtained in two different periods, and this method plays a significant role in the change observation of land use change, flood disaster, earthquake, and fire.

Many remote sensing image change detection methods have been proposed to detect the changed information, and these methods can be divided into two components: supervised and unsupervised algorithms [18,19]. Because the corresponding classifier in supervised change detection method usually needs to be trained with available labeled data, its acquisition usually takes time and is costly. Compared with the supervised method, the unsupervised method does not need labeled reference images for training; in general, the multi-temporal remote sensing images we obtained do not have reference images, which matches the practical applications. Remote sensing image change detection mainly contains three steps: preprocessing (e.g., geometric registration or denoising); difference image generation; and analyzing the difference image to obtain the change detection map.

The thresholding-based, segmentation-based, and clustering-based methods are widely used in unsupervised change detection approaches [15]. In terms of the thresholding-based methods, the Kittler-Illingworth minimum-error thresholding method [20], the Otsu method [21], and likelihood ratio method [22] are used. Gong et al. [23] introduced a synthetic aperture radar (SAR) image change detection method based on a neighborhood-based ratio (NR) operator and the generalization of Kittler and Illingworth thresholding (GKIT) model. Xu et al. [24] proposed SAR image change detection method using a modified neighborhood-based operator and iterative Otsu model. Geetha et al. [25] proposed multi-temporal SAR image change detection using a Laplacian pyramid and Otsu model. For the segmentation-based methods, Celik et al. [26] proposed a remote sensing image change detection method based on an undecimated discrete wavelet transform and Chan–Vese segmentation model. The clustering-based methods are most popular in the image change detection, e.g., Celik et al. [27] introduced one remote sensing image change detection method using a principal component analysis and K-means clustering (PCAKM) model. Li et al. [28] proposed an unsupervised SAR change detection using gabor wavelet and fuzzy C-means clustering. Chen et al. [29] introduced the nonsubsampled contourlet transform-Hidden Markov Tree model (NSCT-HMT) model and fuzzy local information c-means (FLICM) into the remote sensing image change detection. The aforementioned change detection methods have made some achievements in the field of remote sensing change detection. 

In recent studies, the deep learning methods have been successfully applied to remote sensing change detection. These methods include principal component analysis network (PCANet) [30], channel weighting-based deep cascade network [31], convolutional-wavelet neural networks [32], multiscale capsule network [33], transferred deep learning [34], deep pyramid feature learning networks [35], attention-based deeply supervised network [36], etc. Because the methods based on deep learning use training samples for training, the accuracy of the final change detection results is also relatively high.

In this paper, we present a novel remote sensing image change detection method based on a multiscale geometric analysis fusion and FLICM model. Simulation experiments on four groups of remote sensing images verify the practicability and effectiveness of the proposed algorithm.

## 2. Methodology

**Figure 1:**
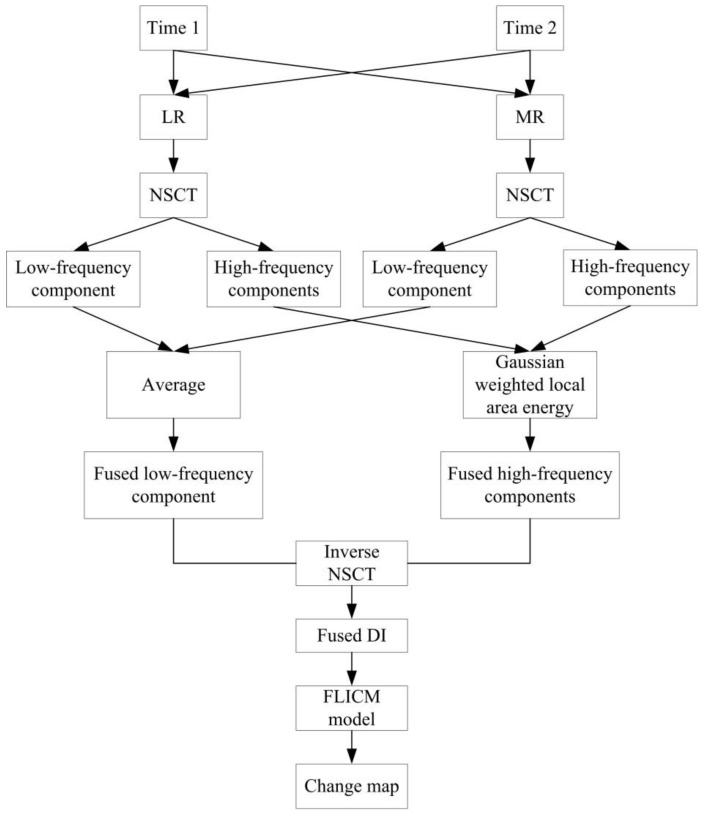
The structure of the proposed remote sensing image change detection algorithm.

This section introduces the proposed remote sensing image change detection method, and we assume that multi-temporal remote sensing images are registered. The main contents include the difference image (DI) calculated by log-ratio operator (LR) and mean-ratio operator (MR), respectively; the fused difference image generated by NSCT fusion; and the final change detection map computed by FLICM model. The structure of the proposed algorithm is shown in Figure 1.

### 2.1. Multiscale Geometric Analysis

Multiscale geometric analysis includes ridgelet, curvelet, contourlet, and shearlet transform, etc. [37]. These transforms have been widely used in image processing, such as image denoising and image fusion. Nonsubsampled contourlet transform (NSCT) is the optimization model of contourlet [38], and it is a translation invariant, multiscale, and multidirectional transformation. NSCT is constructed by a nonsubsampled pyramid (NSP) and nonsubsampled directional filter bank (NSDFB). Firstly, NSP decomposes the input image into high-pass and low-pass parts, and then NSDFB decomposes the high-frequency sub-band into multiple directional sub-bands, and the low-frequency part continues to be decomposed, as above. Liu et al. [39] introduced the image fusion based on NSCT and sparse representation model.

### 2.2. Difference Image Generation 

In the process of remote sensing image change detection, the difference image (DI) generation is an important step. It is assumed that there are registered and corrected remote sensing images *X* and *Y*, and the difference images computed by the log-ratio operator (LR) [40] and mean-ratio operator (MR) [41] are described as follows:

(1) $$LR=\left|log\frac{Y}{X}\right|=\left|logY-logX\right|,\;$$

(2) $$MR=1-min\left(\frac{\mu_{1}}{\mu_{2}},\frac{\mu_{2}}{\mu_{1}}\right),$$

where $\mu_{1}$ and $\mu_{2}$ show the local mean values of the remote sensing images *X* and *Y*, respectively.

The background information generated by the log-ratio image is relatively flat, and the change area information reflected by the mean-ratio image is relatively consistent with the real change trend of the remote sensing image. Therefore, the log-ratio image and mean-ratio image can be integrated into one new difference image with complementary information. Compared with single difference image computed by the log-ratio or mean-ratio operator, the fused difference image can not only reflect the real change trend but also suppress the background.

In order to achieve more useful information, we integrate the two difference images through NSCT transformation. The main step of the NSCT-based fusion can be concluded as follows.

Step 1: The LR and MR images are decomposed by NSCT into low-frequency (LF) and high-frequency (HF) components, respectively. We define them as $\left\{DI_{LF}^{LR},\;DI_{LF}^{MR}\right\}$ and $\left\{DI_{HF}^{LR},\;DI_{HF}^{MR}\right\}$.

Step 2: Fuse the low- and high-frequency components using the average rule and Gaussian weighted local area energy rule, respectively.

(3) $$DI_{LF}^{fuse}=\left(DI_{LF}^{LR}+DI_{LF}^{MR}\right)/2,$$

(4) $$DI_{HF}^{fuse}\left(i,j\right)=\left\{\begin{array}{c} DI_{HF}^{LR}\left(i,j\right),\;E_{HF}^{LR}\left(i,j\right)\leq E_{HF}^{MR}\left(i,j\right)\\ DI_{HF}^{MR}\left(i,j\right),\;E_{HF}^{LR}\left(i,j\right)>E_{HF}^{MR}\left(i,j\right) \end{array}\right.,$$

where $E_{HF}\left(i,j\right)$ shows the Gaussian weighted local area energy coefficient, and it is computed by

(5) $$E_{HF}\left(i,j\right)=\sum_{h=-p}^{p}\sum_{t=-p}^{p}g_{h,t}{\left[DI_{HF}\left(i+h,\;j+h\right)\right]}^{2},$$

where $g_{h,t}$ shows the element of the rotationally symmetric Gaussian low-pass filter ***g*** of size $\left(2p+1\right)\times \left(2p+1\right)$ with standard deviation σ=1.

Step 3: The fused difference image $DI^{final}$ is calculated by the inverse NSCT performing on fused low-frequency $DI_{LF}^{fuse}\;$and high-frequency $DI_{HF}^{fuse}$.

In this section, the NSCT decomposition level is one, and it has one low-frequency sub-band and two high-frequency sub-bands. This ensures the running time of the algorithm and achieves good fusion effect. Subsequently, the fused difference image will be analyzed by the FLICM model.

### 2.3. FLICM Model

In the fuzzy local information c-means (FLICM) clustering model, the fuzzy factor $G_{ki}$ is defined as follows [42]:

(6) $$G_{ki}=\sum_{j\in N_{I}}\frac{1}{d_{ij}+1}{\left(1-u_{kj}\right)}^{m}\|x_{j}-v_{k}{}^{2}\|,\;$$

where the *i*th pixel represents the center of the local window, the *j*th pixel depicts the neighboring pixels falling into the window around the *i*th pixel, and $d_{ij}$ presents the spatial Euclidean distance between pixels *i* and *j*. $v_{k}$ shows the prototype of the center of cluster *k*, and $u_{kj}\;$ shows the fuzzy membership of the gray value *j* with respect to the *k*th cluster. $\left\|x_{j}-v_{k}{}^{2}\right\|$ shows the Euclidean distance between object $x_{j}$ and cluster center $v_{k}$.

According to the previously defined function $G_{ki}$, the objective function of the FLICM model is calculated by

(7) $$J_{m}=\sum_{i=1}^{N}\sum_{k=1}^{c}\left[u_{ki}^{m}\|x_{i}-v_{k}{}^{2}\|+G_{ki}\right],\;$$

where $v_{k}$ and $u_{ki}$ have the same meaning as in Equation (6). *N* and *c* represent the number of the data items and clusters, respectively. $\left\|x_{i}-v_{k}{}^{2}\right\|$ shows the Euclidean distance between object $x_{i}$ and cluster center $v_{k}$. The $u_{ki}$ and $v_{k}$ are defined as follows:

(8) $$u_{ki}=\frac{1}{\sum_{j=1}^{c}{\left(\frac{\|x_{i}-v_{k}\|{}^{2}+G_{ki}}{\|x_{i}-v_{j}\|{}^{2}+G_{ji}}\right)}^{1/\left(m-1\right)}},$$

(9) $$v_{k}=\frac{\sum_{i=1}^{N}u_{ki}^{m}x_{i}}{\sum_{i=1}^{N}u_{ki}^{m}}.$$

## 3. Experimental Results and Discussion

**Figure 2:**
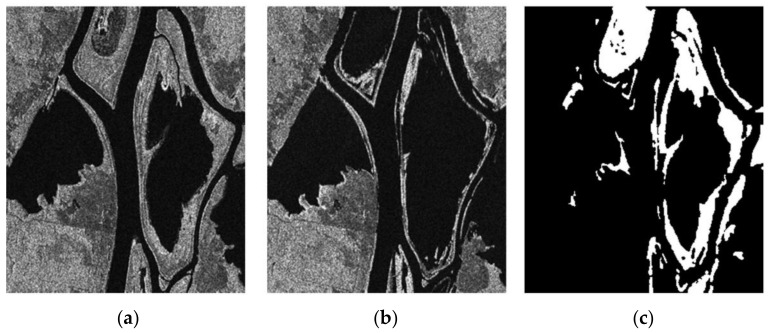
Ottawa data set. (**a**) Image acquired in May 1997; (**b**) image acquired in August 1997; (**c**) reference.

**Figure 3:**
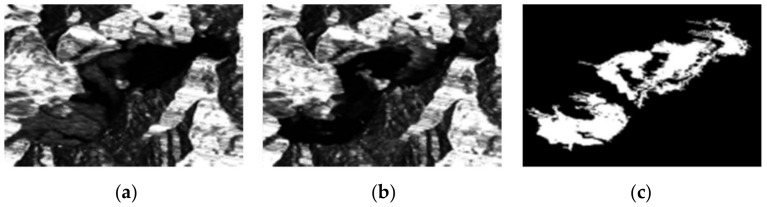
Wenchuan data set. (**a**) Image acquired on 3 March 2008; (**b**) image acquired on 16 June 2008; (**c**) reference.

**Figure 4:**
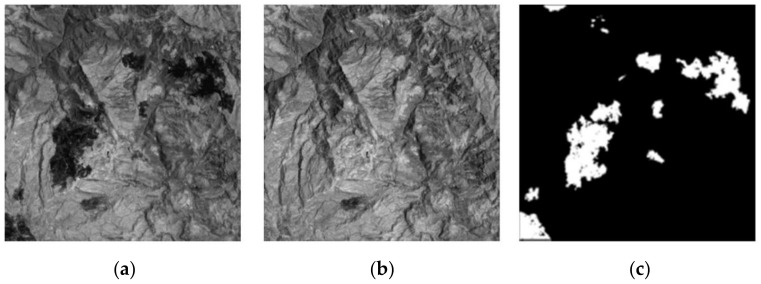
Mexico data set. (**a**) Image acquired in April 2000; (**b**) image acquired in May 2005; (**c**) reference.

**Figure 5:**
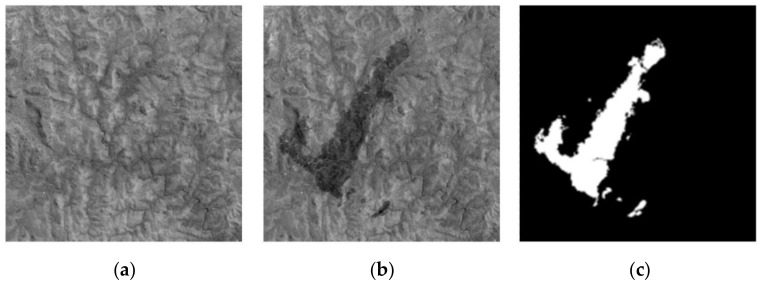
Yambulla data set. (**a**) Image acquired on 1 October 2015; (**b**) image acquired on 6 February 2016; (**c**) reference.

In this section, two groups of SAR images and two groups of optical images are used to simulate. In order to evaluate the detection accuracy of the proposed algorithm more accurately, subjective and objective evaluations are adopted. Some state-of-the-art change detection methods are compared, such as PCAKM [27], Gabor wavelet and two-level clustering (GaborTLC) [28], LMT [43], PCANet [30], NRELM [44], neighborhood-based ratio and collaborative representation (NRCR) [45], and convolutional-wavelet neural networks (CWNN) [32]. Meanwhile, the false negative (FN) [32], false positive (FP) [32], overall error (OE) [32], percentage correct classification (PCC) [32], kappa coefficient (KC) [32,46,47], and F1-score (F1) [18] are used as the objective evaluation metrics. Figure 2, Figure 3, Figure 4 and Figure 5 show the remote sensing images for simulating. 

### 3.1. Experimental Data

The first data utilized in the experiment is the Ottawa data set with the size 290 × 350 pixels. The original images were obtained in May and August 1997, respectively, which are shown in Figure 2a,b. The corresponding ground-truth image is depicted in Figure 2c. 

The second data is the Whenchuan data set with the size 442 × 301 obtained by ESA/ASAR on 3 March 2008 and 16 June 2008, respectively, which are shown in Figure 3a,b. The corresponding reference image is shown in Figure 3c.

The third data is the Mexico data set of optical images with the size 512 × 512 captured in April 2000 and May 2005, respectively. The two original images and the reference image are depicted in Figure 4. 

The fourth data is the Yambulla data set consists of two optical images with the size of 500 × 500 pixels (as shown in Figure 5); they were acquired on 1 October 2015 and 6 February 2016 over the area of the Yambulla State Forest (Australia), respectively. More details of the data sets are concluded in Table 1.

### 3.2. Analysis of the Difference Image

**Figure 6:**
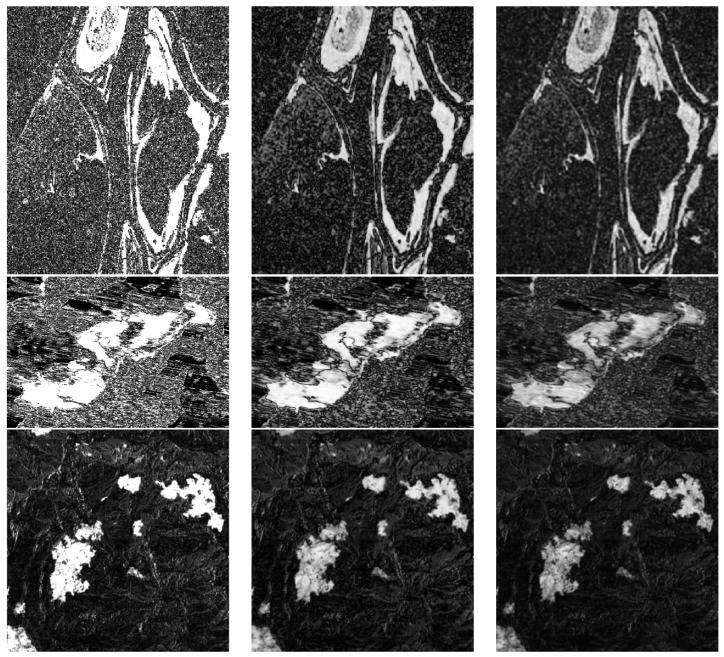

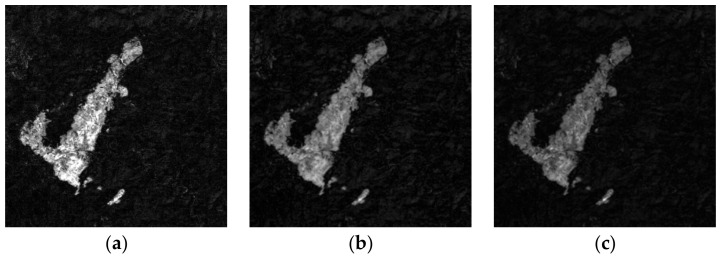
The difference images with different methods. (**a**) Log-ratio operator; (**b**) mean-ratio operator; (**c**) NSCT fusion.

In this subsection, we discuss the difference images generated by different methods and the change detection results generated by FLICM model. In Figure 6, we can see the difference images computed by the log-ratio operator (LR), mean-ratio operator (MR), and NSCT fusion, respectively. 

**Figure 7:**
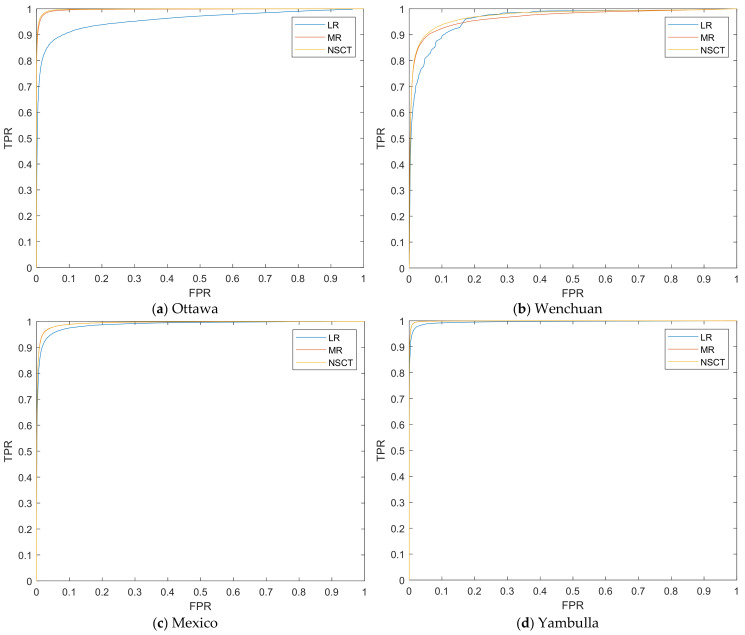
The ROC curves of operators generated DIs. (**a**) Ottawa; (**b**) Wenchuan; (**c**) Mexico; (**d**) Yambulla.

The performance of the difference images (DIs) computed by the LR, MR, and NSCT fusion models are evaluated by the empirical receiver operating characteristics (ROC) curves (as shown in Figure 7), which are plotted by utilizing the true positive (TP) rate (TPR) versus the false positive (FP) rate (FPR). Moreover, two quantitative criteria derived from the ROC curve can be calculated: the area under the curve (AUC) [48] and the diagonal distance (Ddist) [48], as well as the corresponding metrics, are shown in Table 2. For the two metrics, the larger the criterion, the better the detection. From Table 2, we can denote that the NSCT fusion model performs better than the LR and MR operators.

**Figure 8:**
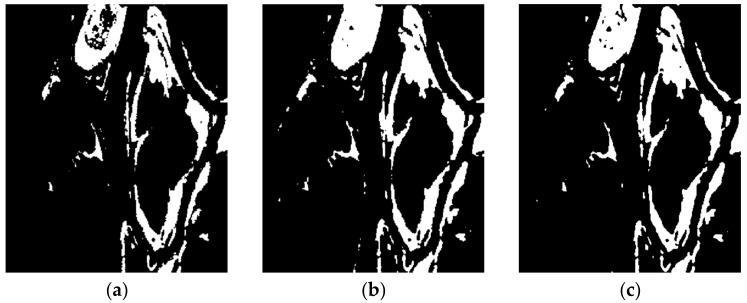
The change detection results with FLICM model. (**a**) LR_FLICM; (**b**) MR_FLICM; (**c**) NSCT_FLICM.

Figure 8 shows the change detection results of the difference images with the FLICM model on Ottawa data set, and the corresponding metrics data are shown in Table 3. Figure 8a has the high alarm missing rate; in other words, FN value is too large; Figure 8b has the high false detection rate, and the FP is large; Figure 8c is the best change detection result, with the highest values of PCC, KC, and F1; at the same time, the balanced FN and FP values are generated, and it has the lowest OE value. This also shows that the result of fused difference image computed by the proposed method is better than that of single LR and MR images.

### 3.3. Experimental Comparison

**Figure 9:**
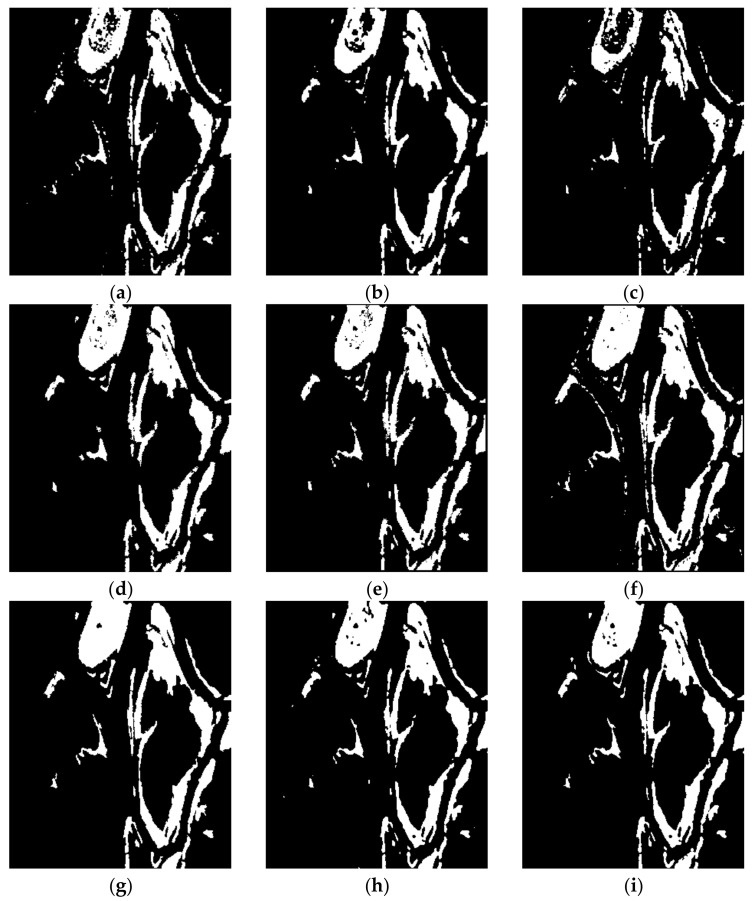
The results of different methods on Ottawa data set. (**a**) PCAKM; (**b**) GaborTLC; (**c**) LMT; (**d**) PCANet; (**e**) NRELM; (**f**) NRCR; (**g**) CWNN; (**h**) proposed method; (**i**) reference.

**Figure 10:**
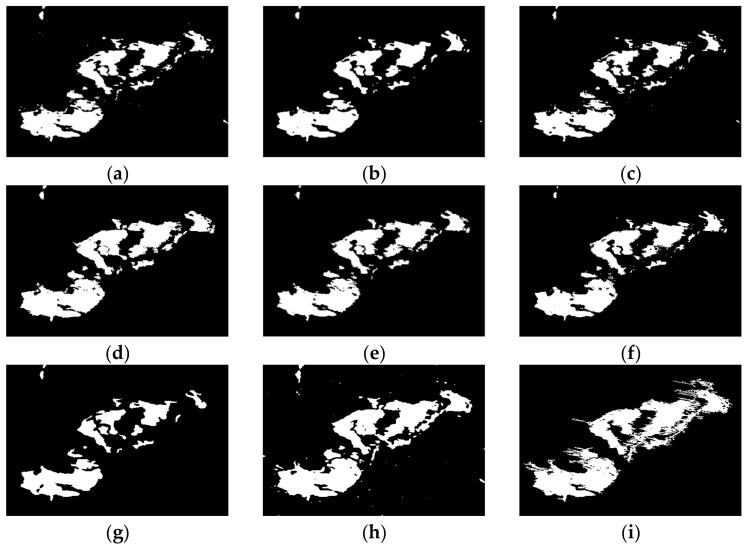
The results of different methods on Wenchuan data set. (**a**) PCAKM; (**b**) GaborTLC; (**c**) LMT; (**d**) PCANet; (**e**) NRELM; (**f**) NRCR; (**g**) CWNN; (**h**) proposed method; (**i**) reference.

**Figure 11:**
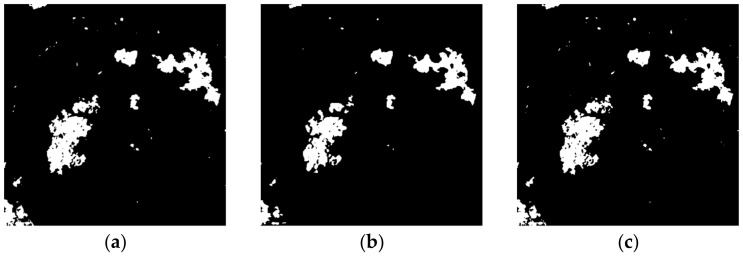

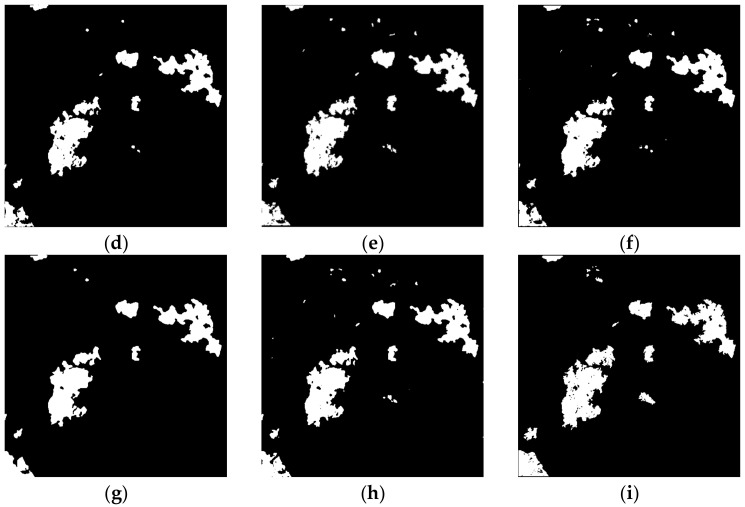
The results of different methods on Mexico data set. (**a**) PCAKM; (**b**) GaborTLC; (**c**) LMT; (**d**) PCANet; (**e**) NRELM; (**f**) NRCR; (**g**) CWNN; (**h**) proposed method; (**i**) reference.

**Figure 12:**
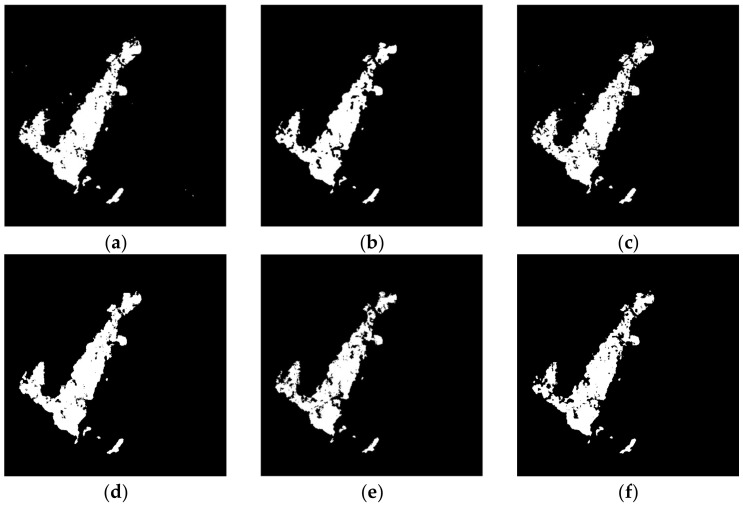

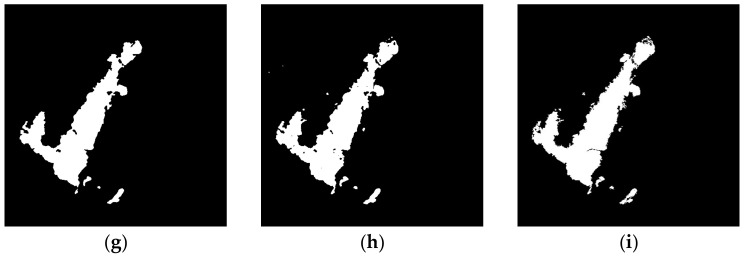
The results of different methods on Yambulla data set. (**a**) PCAKM; (**b**) GaborTLC; (**c**) LMT; (**d**) PCANet; (**e**) NRELM; (**f**) NRCR; (**g**) CWNN; (**h**) proposed method; (**i**) reference.

The change detection results generated by the proposed remote sensing image change detection algorithm, as well as seven comparative approaches, are depicted in Figure 9, Figure 10, Figure 11 and Figure 12 and Table 4, Table 5, Table 6 and Table 7. 

Figure 9 shows the change maps on Ottawa data set. From the results, it can be seen that the LMT method generates the worst performance, and it has the highest FN value. The PCAKM and GaborTLC methods have high missed detection, losing some detail information. The NRCR method has more false detection, exhibiting many isolated spots with the highest FP values. The PCANet and NRELM algorithms give a similar performance, but these two methods still have some missed detection with high FN value. The visual performance obtained by CWNN technique is better than the previously mentioned six algorithms, while it has some false detection with high FP value. For the proposed change detection model, it achieves the best performance compared to other state-of-the-art approaches, and the change map is closer to the reference image. Table 4 gives the FN, FP, OE, PCC, KC, and F1 values for the different image change detection algorithms on the Ottawa data set, respectively. The proposed method achieves the best OE, PCC, KC, and F1 values, and these values are consistent with the visual effect of the experiment.

Figure 10 shows the change results on Wenchuan data set, and the corresponding quantitative evaluation is given in Table 5. From the results, it can be observed that the PCAKM, GaborTLC, LMT, PCANet, NRELM, NRCR, and CWNN methods have high missed detection, and they have high FN values, especially in the CWNN model, and the FN is the highest. Compared to other approaches, the change detection result obtained by the proposed method is the best, the balanced FN and FP values are generated, it matches the reference image best. From Table 5, we can conclude that the FN, OE, PCC, KC, and F1 values achieved by the proposed technique are the best, and the FP value generated by the CWNN model is the best. KC is a more comprehensive evaluation metric, and the KC value of the proposed method is 8.24%, 11.24%, 15.40%, 3.47%, 5.99%, 9.49%, and 16.71% ahead of PCAKM, GaborTLC, LMT, PCANet, NRELM, NRCR, and CWNN, respectively.

Figure 11 and Table 6 give the results on Mexico data set. From the results, it can be seen that the PCAKM, GaborTLC, LMT, and PCANet approaches have high missed detection, the corresponding FN values are high, and the FN value achieved by GaborTLC is the highest. The NRELM, NRCR, and CWNN techniques achieve better performance compared to aforementioned four methods. The result generated by the proposed technique has the highest visual effect advantage compared to the state-of-the-art methods. From the data as shown in Table 6, we can see that the FN, OE, PCC, KC, and F1 values generated by our method are the best, and the FP value achieved by the GaborTLC method is the best. The KC value of the proposed algorithm is 4.69%, 12.07%, 5.27%, 3.03%, 0.37%, 1.08%, and 2.57% ahead of PCAKM, GaborTLC, LMT, PCANet, NRELM, NRCR, and CWNN, respectively. Qualitative and quantitative evaluations of this group of experiments have achieved consistency.

Figure 12 depicts the change maps on Yambulla data set. From the results, it can be seen that the GaborTLC, LMT, NRELM, and NRCR techniques suppress the noise, and the false detection rate is reduced, while they have high missed detection. The PCAKM, PCANet, and CWNN methods generate better performance, but the missed detection rate is still high. Compared with other seven algorithms, the change map generated by our method is the best, and it has the lowest missed detection rate. From the data as shown in Table 7, the values of FN, OE, PCC, KC, and F1 achieved by the proposed method are the best. The KC value of the proposed method is 2.58%, 10.61%, 6.54%, 5.17%, 14.07%, 11.28%, and 1.86% ahead of PCAKM, GaborTLC, LMT, PCANet, NRELM, NRCR, and CWNN, respectively. The qualitative and quantitative evaluations of this group of data are consistent, which proves the superiority of our algorithm.

**Figure 13:**
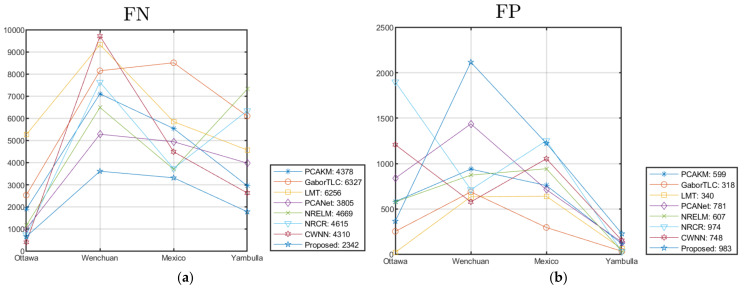

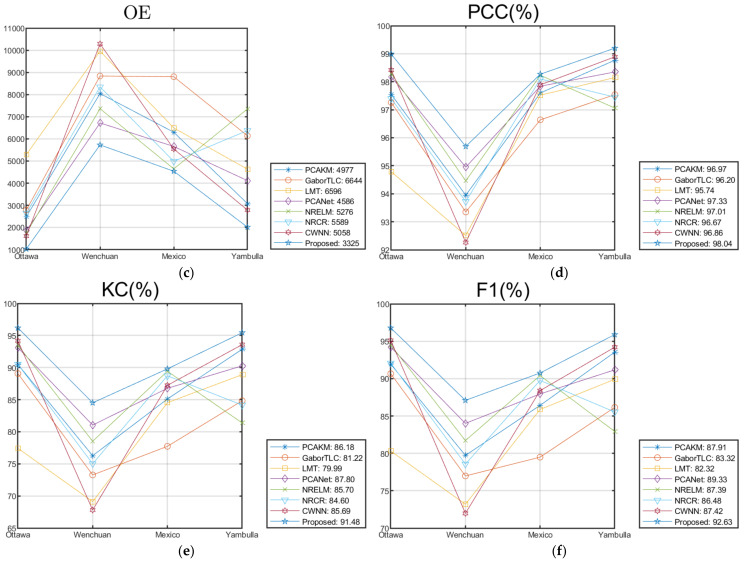
Objective performance of the methods on different data sets. (**a**) FN; (**b**) FP; (**c**) OE; (**d**) PCC; (**e**) KC; (**f**) F1.

In order to verify the effectiveness and superiority of the proposed algorithm more accurately, we take the average value of the simulation experimental data of four groups of remote sensing images, as shown in Table 8. The index value distribution fluctuation line of each group of data and comparison algorithms are shown in Figure 13, and the average values are given in the legend. From Table 8, we can denote that the scores of FN, OE, PCC, KC, and F1 generated by the proposed method are the best. The effectiveness of the proposed algorithm is objectively proved.

## 4. Conclusions

In this paper, a novel remote sensing image change detection method based on NSCT fusion and FLICM model is proposed. The background information computed by the log-ratio image is relatively flat, and the change area information reflected by the mean-ratio image is relatively consistent with the real change trend of the remote sensing image. Therefore, the log-ratio image and mean-ratio image can be integrated into one new difference image with complementary information. Based on these analysis, the difference images generated by log-ratio and mean-ratio operators are fused by the NSCT model, and the fused difference image is obtained. Then, the FLICM model is used to generate the final change detection map. We carried out simulation experiments on four groups of remote sensing images. The experimental results verify the effectiveness of our algorithm by qualitative and quantitative evaluations with other algorithms. Our method can be effectively applied to land cover, flood, earthquake, and forest fire monitoring. In our experiment, we only simulate and verify the change detection in homogeneous remote sensing images. In future work, we will explore and improve the proposed algorithm for change detection in heterogeneous remote sensing images.

## Figures and Tables

**Table 1:** The description of the four data sets used in the experiment.

Scenario (Data Set)	Location	Data	Event	Size	Satellite	Sensor Type
1	Ottawa, Canada	May 1997 August 1997	Flood	290 × 350	Radarsat-1	SAR
2	Wenchuan, China	3 March 2008 16 June 2008	Earthquake	442 × 301	Radarsat-2	SAR
3	Mexico	April 2000 May 2005	Fire	512 × 512	Landsat-7	Optical
4	Yambulla, Australia	1 October 2015 6 February 2016	Bushfire	500 × 500	Landsat-8	Optical

**Table 2:** The quantitative criteria AUC and Ddist of different operators on remote sensing image data sets.

Methods	Ottawa		Wenchuan		Mexico		Yambulla	
	AUC	Ddist	AUC	Ddist	AUC	Ddist	AUC	Ddist
LR	0.9573	1.2829	0.9618	1.2701	0.9877	1.3467	0.9954	1.3815
MR	0.9969	1.3828	0.9665	1.2953	0.9937	1.3689	0.9987	1.3980
NSCT	0.9980	1.3857	0.9729	1.3063	0.9938	1.3681	0.9990	1.3986

**Table 3:** The objective evaluations of change detection on Ottawa in Figure 8.

	FN	FP	OE	PCC (%)	KC (%)	F1 (%)
LR_FLICM	2588	224	2812	97.23	88.93	90.54
MR_FLICM	340	896	1236	98.78	95.49	96.21
NSCT_FLICM	658	366	1024	98.99	96.18	96.78

**Table 4:** The objective evaluations of change detection on Ottawa in Figure 9.

	FN	FP	OE	PCC (%)	KC (%)	F1 (%)
PCAKM	1901	582	2483	97.55	90.49	91.93
GaborTLC	2531	253	2784	97.26	89.07	90.66
LMT	5266	23	5289	94.79	77.43	80.31
PCANet	1011	839	1850	98.18	93.12	94.21
NRELM	1157	578	1735	98.29	93.48	94.50
NRCR	739	1900	2639	97.40	90.51	92.07
CWNN	399	1208	1607	98.42	94.17	95.12
Proposed	658	366	1024	98.99	96.18	96.78

**Table 5:** The objective evaluations of change detection on Wenchuan in Figure 10.

	FN	FP	OE	PCC (%)	KC (%)	F1 (%)
PCAKM	7111	939	8050	93.95	76.27	79.73
GaborTLC	8155	688	8843	93.35	73.27	76.98
LMT	9333	635	9968	92.51	69.11	73.19
PCANet	5284	1437	6721	94.95	81.04	84.01
NRELM	6492	873	7365	94.46	78.52	81.71
NRCR	7638	713	8351	93.72	75.02	78.56
CWNN	9720	578	10298	92.26	67.80	71.97
Proposed	3612	2117	5729	95.69	84.51	87.09

**Table 6:** The objective evaluations of change detection on Mexico in Figure 11.

	FN	FP	OE	PCC (%)	KC (%)	F1 (%)
PCAKM	5543	759	6302	97.60	85.11	86.42
GaborTLC	8515	296	8811	96.64	77.73	79.49
LMT	5855	640	6495	97.52	84.53	85.87
PCANet	4946	713	5659	97.84	86.77	87.95
NRELM	3702	943	4645	98.23	89.43	90.41
NRCR	3734	1252	4986	98.10	88.72	89.76
CWNN	4491	1053	5544	97.89	87.23	88.39
Proposed	3316	1223	4539	98.27	89.80	90.75

**Table 7:** The objective evaluations of change detection on Yambulla in Figure 12.

	FN	FP	OE	PCC (%)	KC (%)	F1 (%)
PCAKM	2956	116	3072	98.77	92.86	93.54
GaborTLC	6105	34	6139	97.54	84.83	86.15
LMT	4571	60	4631	98.15	88.90	89.91
PCANet	3979	134	4113	98.35	90.27	91.17
NRELM	7325	33	7358	97.06	81.37	82.93
NRCR	6348	31	6379	97.45	84.16	85.53
CWNN	2629	153	2782	98.89	93.58	94.20
Proposed	1782	227	2009	99.20	95.44	95.89

**Table 8:** The average objective evaluations of change detection on the four data sets.

	FN	FP	OE	PCC (%)	KC (%)	F1 (%)
PCAKM	4378	599	4977	96.97	86.18	87.91
GaborTLC	6327	318	6644	96.20	81.22	83.32
LMT	6256	340	6596	95.74	79.99	82.32
PCANet	3805	781	4586	97.33	87.80	89.33
NRELM	4669	607	5276	97.01	85.70	87.39
NRCR	4615	974	5589	96.67	84.60	86.48
CWNN	4310	748	5058	96.86	85.69	87.42
Proposed	2342	983	3325	98.04	91.48	92.63

## Data Availability

Not applicable.

## Abbreviations

NSCT	Nonsubsampled contourlet transform
FLICM	Fuzzy local information C-means clustering
DI	Difference image
SAR	Synthetic aperture radar
NR	Neighborhood-based ratio
GKIT	Generalization of Kittler and Illingworth thresholding
PCAKM	Principal component analysis and K-means clustering
NSCT-HMT	Nonsubsampled contourlet transform-Hidden Markov Tree
PCANet	Principal component analysis network
LR	Log-ratio
MR	Mean-ratio
NSP	Nonsubsampled pyramid
NSDFB	Nonsubsampled directional filter bank
LF	Low-frequency
HF	High-frequency
GaborTLC	Gabor wavelet and two-level clustering
LMT	Logarithmic mean-based thresholding
NRELM	Neighborhood-based ratio and extreme learning machine
NRCR	Neighborhood-based ratio and collaborative representation
CWNN	Convolutional-wavelet neural networks
FN	False negative
FP	False positive
OE	Overall error
PCC	Percentage correct classification
KC	Kappa coefficient
F1	F1-score
ROC	Receiver operating characteristics
TPR	True positive rate
FPR	False positive rate
AUC	Area under the curve
Ddist	Diagonal distance
